# ‘True Day Case' Laparoscopic Cholecystectomy in a High-Volume Specialist Unit and Review of Factors Contributing to Unexpected Overnight Stay

**DOI:** 10.1155/2018/1260358

**Published:** 2018-07-24

**Authors:** A. Solodkyy, A. R. Hakeem, N. Oswald, F. Di Franco, S. Gergely, A. M. Harris

**Affiliations:** Department of Upper Gastrointestinal Surgery, Hinchingbrooke Hospital, Hinchingbrooke Park, Huntingdon, Cambridgeshire PE29 6NT, UK

## Abstract

**Introduction:**

Laparoscopic cholecystectomy (LC) is the gold standard treatment for gallstones. British Association of Day Case Surgery recommends at least 60% of LCs be performed as day cases. The aim of this study was to assess our rate of true day case LCs and review factors preventing same-day discharge.

**Methods:**

We prospectively collected data of all elective LCs performed in a district general hospital over 32 months.

**Results:**

500 patients underwent LC during this period; 438 (88.2%) patients were planned day cases and 59 patients (11.8%) planned overnight stays. Of the planned day cases, 75.8% (n=332) were discharged on the same day and 106 (24.2%) had unexpected overnight stay (UOS). Most patients with BMI >35 and ASA3 planned day case patients were successfully discharged. Drain insertion, longer operations, and late recovery departure were the main reasons for UOS. There were more complications in this group compared to day cases.

**Conclusions:**

This unit has a high ‘true day case' rate of 75.8%. High BMI and ASA3 should not be absolute contraindications to day case surgery. The majority of unexpected overnight stays are unavoidable but may be reduced by patient selection, stringent preoperative assessment, operation scheduling, and reduction in unnecessary drain insertion.

## 1. Introduction

Laparoscopic cholecystectomy (LC) is the gold standard of the treatment for gallstones [1]. Increasing the number of elective LCs performed as day case (at least 75%) is a key target in the National Health Service (NHS) plan issued by the UK Department of Health [2]. British Association of Day Surgery (BADS) recommends that at least 60% of LC operations should be performed as day cases both for optimal patient outcomes and cost-effectiveness [3]. Although a number of studies have reported that day case LC is feasible, effective, and safe, the national average for successful day case LC is reported to be only around 16% [3–6]. This may be in part due to use of so-called ‘absolute criteria' guiding admission policies in many units.

There is still ambiguity in the way the day case LC data is presented in the literature, with considerable confusion between the so-called ‘23-hour' stay and true day cases where the patient is discharged on the same day of surgery [7, 8]. The definition of ‘true day case' procedure as per BADS guidelines is as follows: a patient is admitted, has a procedure performed, requires recovery facilities, and is discharged on the same day and does not stay overnight in the hospital [3]. However, some units ‘reinterpret' the definition and hence report those patients who stayed overnight and get discharged the following morning as ‘day cases', based on the 23-hour stay guideline [7].

Unexpected admission following day case procedure is an unwanted outcome for the patient, the surgeon, and the trust [9, 10]. Overnight stay leads to inconvenience among the patients and carers and can lead to poor patient experience and satisfaction [11]. Admission of these patients increases the pressure on acute hospital beds and has significant economic implications. A high unexpected admission rate invariably reflects on the suboptimal efficiency of the trust and is considered as a failure of day care facilities [12, 13].

The aims of this study were to determine the rate of ‘true day case' LCs in a high-volume setting and also to study the factors that lead to unexpected admissions following LC.

## 2. Methods

We prospectively collected data for all patients who underwent day case LC over a period of 32 months between March 2010 and October 2012. The Upper Gastrointestinal Unit at Hinchingbrooke Hospital performs around 300 LCs per year, of which 75-80% are elective planned admissions and the remainder done as emergencies. All patients for day case LC were listed following review in outpatient clinic or after emergency admission without requiring urgent surgery (when symptoms settled rapidly allowing discharge and a planned elective procedure). In every case gallstones were confirmed on ultrasound scan.

The patient inclusion and exclusion criteria for day case LC were based on agreed local trust guidelines on day case surgery and include three categories: social factors, medical factors, and surgical factors. The social factor includes their understanding of day case procedure, their social support for the first 24 hours, and domestic circumstances for postoperative care. The medical factors, such as their comorbidities and contraindications to day surgery, were reviewed by the consultant anaesthetists or specialist nurses, who had the final say on listing for day case or planned admission. The listing surgeon decides on the appropriateness of the laparoscopic procedure, including the risk of serious complications, recovery postprocedure, and mobility.

Our only absolute criterion for allocation to the overnight admission group is social, i.e., those patients who do not have a responsible adult at home to look after them for 24 hours postprocedure. The other criteria such as American Society of Anaesthesiology (ASA) class 3 and above, body mass index (BMI) above 35, previous severe pancreatitis, and previous extensive abdominal surgery are considered to be relative contraindications to being listed as day case procedure; while some of these patients inevitably require overnight stay, many are routinely planned as day cases. Patients with multiple comorbidities are all assessed and treated in this unit and not referred to a tertiary centre. Diabetic patients are routinely placed first on the morning list and usually discharged on the same day. Patients on anticoagulation present a range of clinical risk: those on prophylaxis for uncomplicated atrial fibrillation can often be successfully treated as day cases whereas those with higher risk disease (e.g., coronary stents or recurrent DVTs) are usually planned for overnight stay with advice sought where appropriate from cardiologist and/or anticoagulant nurse specialists. A diagnosis of common bile duct (CBD) stones elevates the complexity of the case; if preoperative endoscopic retrograde cholangiopancreatography (ERCP) successfully clears the duct, the patient may still be considered for day case surgery, but planned laparoscopic CBD exploration will inevitably require inpatient care.

Patients were admitted to the day case unit at 07:30 if their operation was scheduled for the morning list and 11:00 if scheduled for the afternoon. The patients wore thromboembolic deterrent (TED) stockings unless contraindicated. All patients had antibiotic prophylaxis at induction and intraoperative temperature was maintained by warming blanket. All patients underwent standard LC using four-port technique. Intra-abdominal CO_2_ insufflation pressure was set at 10-12mmHg. The port sites were injected with 20ml of 0.25% bupivacaine. Drains were not placed routinely but used selectively where surgically indicated, e.g., after particularly difficult dissection with unexpected bleeding. This unit operates a selective policy for intraoperative cholangiography, depending on preoperative bloods and imaging; rarely it is required for difficult anatomy.

All patients were operated on by a consultant, or experienced registrar with consultant available for advice. Junior trainees not yet independent on this operation were supervised by the consultant scrubbed in theatre.

Postoperatively patients were transferred to recovery and then back to the day case ward, from where they could be discharged until 22:00 if they met day case unit criteria for discharge shown in the appendix. If there are any contraindications to discharge, they were admitted for overnight stay.

All patients were followed up by telephone at 4–6 weeks postoperatively by a surgical care practitioner who completed a follow-up questionnaire and collected outcome data prospectively including any postoperative complications such as wound infection, haematoma, and readmissions.

The data collected for this study included demographic details and operative details including timing of the procedure (AM or PM list), length of operation, use of on-table cholangiogram, insertion of drains, intraoperative complications, time of leaving theatre recovery, grade of operating surgeon, and any immediate postoperative complications. The reasons for overnight admission were categorised as pain control, nausea and vomiting, drain in situ, urinary retention, other anaesthetic and social issues, positive on-table cholangiogram (if there is a need for urgent postoperative ERCP), long operation, and late-evening completion of the procedure. Long operation was defined as 90 minutes or longer and late operation was defined as leaving recovery later than 18:30. Leaving recovery after this time would not allow 4 hours of recovery time before closure of the day case unit at 22:00.

### 2.1. Statistical Analysis

Categorical variables are expressed as the frequency and proportions (%), and continuous variables are presented as the mean ± standard deviation. Categorical variables were analysed by Fisher's exact or chi-square test and continuous variables by unpaired Student's* t*-test or one-way ANOVA (Analysis of Variance). P value <0.05 was considered significant.

The variables which showed statistical significance (p value <0.01) on univariate analysis were further subjected to multivariate analysis using logistic regression model, and odds ratio with 95% confidence interval was calculated. The variables significant on multivariate analysis (p value <0.05) were considered as independent prognosticators of unexpected overnight stay. All statistical analyses were carried out using SPSS for Windows version 20.0 (SPSS Inc., Chicago, IL, USA).

## 3. Results

During the study period, 500 patients underwent elective LC in our unit. Three patients were excluded from the study because they required CBD exploration; therefore 497 cases were analysed. Among those were 391 females and 106 males (ratio 4:1). The median age was 52 with a range of 16-85 years. The median body mass index (BMI) was 29 kg/m^2^ with a range of 17-50 kg/m^2^. The ASA status was ASA 1= 192 (38.6%), ASA 2= 266 (53.5%), and ASA 3= 39 (7.9%). Fifty-nine (11.8%) patients were planned for postoperative overnight stay due to either social or medical reasons. Therefore, overall 438 (88.2%) patients were planned for day case LCs, of which 332 (75.8%) were successfully completed as day case (true day case; TDC) and 106 (24.2%) patients stayed overnight due to various reasons (unexpected overnight stay; UOS).

## 4. Demographics: TDC versus UOS

Those patients who stayed overnight following day case LC were older (UOS; 54.7±15.0 years) when compared to TDC group (49.8±15.6 years; P=0.005). There was no difference between the two groups in terms of gender and BMI distribution; 76% patients with BMI >35 who were planned day cases were discharged on the day of surgery. There were significantly more ASA 2 patients in UOS group, when compared to TDC group (65.1% versus 49.1%; P=0.005). As expected, the TDC group had significantly more fit and healthy ASA 1 status patients (46.4%) when compared to UOS group (28.3%; P=0.001) (Table 1). Of the 22 ASA 3 patients who were planned day cases, 68% were successfully discharged on the day of surgery.

## 5. Reasons for Unexpected Overnight Admissions

The reasons for unexpected overnight admissions are listed in Table 2. Some of the patients stayed overnight due to multiple reasons. Twenty-six (24.6%) patients had drain inserted which was the main reason for UOS. The other common reasons for UOS was leaving theatre recovery after 18:30 (19.8%), anaesthetic/social issues such as prolonged postoperative nausea and vomiting or unanticipated lack of support at home (19.8%), and procedures lasting more than 90 minutes (17.9%).

## 6. Factors Contributing to UOS

There were significantly more patients aged more than 50 years in UOS group when compared with the TDC group (64.1% versus 46.7%; P=0.001). The mean operating time was also longer in the UOS group, which explains why there was unexpected overnight admission in this group (82.5 versus 64.9 mins; P<0.001). The LC took more than 90 minutes in 35.8% of patients in the UOS group, whereas only 12.9% in the TDC group had procedure which was longer than this time (P<0.001). As expected, significantly more patients in the UOS group had OTCs performed (35.8% versus 25.3%; P=0.046) and these were more common when LC took more than 90 minutes (15.1% versus 4.5%; P<0.001). Similarly, 24.5% of patients in the UOS group had drain inserted, whereas no patients (0.0%) in the TDC group had drains (P<0.001).

The majority of patients in the UOS group were operated in the PM list (69.8%) when compared to the TDC group (49.7%; P<0.001). This also explains why significant proportion of UOS patients left recovery after 18:30 when compared to those who were discharged on the same day (22.6% versus 1.8%; P<0.001).

There was no difference in the intraoperative complications between the two groups; however, immediate postoperative complications such as bleeding from the port site and urinary retention were significantly higher in the UOS group (6.6% versus 0.0%; P<0.001). There was no difference in the number of LCs performed by the trainee registrar or staff grade between the TDC and UOS groups, respectively (18.1% versus 11.3%; P=0.131).

On multivariate analysis, age more than 50 years [OR 2.1 (95% CI 1.15-3.78); P=0.015], mean operating time [OR 1.0 (95% CI 1.00-1.04); P=0.017], leaving theatre recovery after 18:30 [OR 15.1 (95% CI 5.41-42.34); P<0.001)], and PM list [OR 2.4 (95% CI 1.30-4.61); P=0.005] were independent prognosticators contributing to unexpected overnight admissions following LC (Table 3).

There was no conversion to open procedure or reoperation during the study period in either group and there was also no bile leak or bile duct injury. There was no difference between the TDC and UOS groups in terms of number of readmissions following discharge [9 (2.7%) versus 2 (1.9%); P=1.000] (Table 4).

## 7. Discussion

It has long been established that LC can be performed safely and effectively as a day case procedure. However, this has usually applied to a selected group of patients using rigid exclusion criteria and thus results in an overall low rate of true day case surgery with implications for procedure cost and bed availability. Many units still routinely book high BMI patients, diabetic patients, and all those classified as ASA3 for an overnight stay.

In this study in a high-volume LC setting in a specialist DGH unit, three-quarters (75.8%) of our planned day case patients were discharged on the same day, which gives an excellent ‘true day case' rate which is significantly higher than previous reports in the literature [5, 8, 9, 11, 13–19].

Day case LC has been shown to be safe, effective, and highly economical. The Cochrane review published in 2013 pooled data from six trials involving 492 patients undergoing LC, with 239 patients in the day case and 253 in the overnight stay group. The review reported no significant difference in postoperative pain scores on visual analogue scale, serious adverse events, hospital readmission rate, time to return to normal activity, return to work, and quality of life after LC. They concluded that day case LC is just as safe as overnight stay LC. However day case discharge did not seem to result in improvement in any patient-oriented outcomes such as return to normal activity or earlier return to work [19].

In a large sample size we have demonstrated that 75.8% patients can be discharged home safely on the day of LC. A number of small studies have demonstrated high rates of compliance with day case LC; however, most of them involve discharge within 23 hours of surgery, which actually means that the patient stays overnight and is discharged home the following morning. Jain et al. reported 269 patients over a 5-year period who underwent LC, of which 79% were discharged home within 8 hours of surgery, 95% were discharged on the same day, and 5% were admitted overnight. The patients in this study were highly selected with only 26% of all patients listed for day case LC, whereas we listed 88.2% of all patients for day case LC. Also the majority of patients in their study were ASA 1 (88%) and the remainder were ASA 2 (12%), there were no intraoperative cholangiograms, and 16% of the patients who stayed more than 8 hours were temporarily transferred to the main hospital surgical ward. Although the results from this study are commendable, any usage of general surgical non-day case bed following LC should be considered as failure of day case intention [20]. Similarly, a number of studies have demonstrated that LC can be performed as day case with careful patient selection. Metcalfe et al. reported no difference between the outcomes when comparison was made between day case LC patient selection with and without strict exclusion criteria such as thickness of gallbladder wall, patient age more than 55 years, and previous sphincterotomy [21], but this will inevitably result in higher rates of planned inpatient admission [7, 22, 23].

Many centres employ stringent or ‘absolute' criteria for selection of patients for day case LC [21, 24, 25]. The common exclusion criteria are age more than 70 years, BMI > 35 kg/m^2^, ASA grade 3 or more, complicated biliary disease (evidence of common bile duct stones or previous endoscopic retrograde cholangiogram, severe pancreatitis, and recurrent admissions with cholecystitis), previous extensive abdominal surgery, or a history of severe postoperative nausea and vomiting [8, 26]. Tescani et al. published a systematic review of 20 randomised controlled trials published on day case LCs between January 2000 and June 2008. Out of 20 trials, only one trial included patients of ASA grade III for day case LC, and only three trials included patients with high BMI (35 or more) for day case LC [24]. Our only absolute exclusion criterion was the absence of a responsible adult to stay with the patient following discharge. We suggest criteria other than social should be considered relative and selection should be done on a case by case basis. We have also demonstrated that the majority of patients with BMI > 35 kg/m^2^ can be planned and successfully completed as day cases and the same applies to patients older than 50 years and ASA grade 3.

On multivariate analysis we identified patient's age more than 50 years, longer length of the procedure, PM operating list, and patient leaving theatre recovery after 18:30 as the independent prognosticators for unexpected admission following day case LC. Some of these factors such as age of the patient and length of the procedure cannot be altered. The prolonged procedure is mostly multifactorial, and in our cohort, it is possibly related to the time taken for OTC, difficult operation, intraoperative complication, and procedures requiring drain insertion. Hence, we recommend that procedures longer than 90 minutes should not be a contraindication for same-day discharge. Previous studies have reported longer operating durations when trainees were involved [27]. However, the longer operating times in our cohort were not related to inexperience of the surgeon, as only two of the 38 prolonged operations were operated by trainee surgeons.

Patients more than 50 years and those who are at risk of having prolonged surgery such as previous cholecystitis, ERCP procedure, and/or severe pancreatitis, high BMI (>35 kg/m^2^), and previous abdominal surgery can be listed for AM rather than PM procedure. Appropriate list selection for these patients could make a substantial difference to the patient satisfaction and true day case rates, which in turn will have significant positive financial implications for the trust and also increase bed availability for emergency admissions at a time when this is sorely needed. Length of the operation and timing of the list have similarly contributed to overnight admission in other published series [6, 28]. Our readmission rate of 2.5% compares favourably with those reported by other centres, and there was no difference in readmission when patients were discharged home on the stay day or had overnight/further stay [28].

Drain insertion after LC is another aspect we have been auditing to see if the rates can be reduced. In this unit we do not insert drains routinely but only in difficult procedures, e.g., those with sepsis or significant intraoperative bleeding. Another option would be to send patients home with drain in situ, which can be monitored and removed by the community nurses. However, this is not without risk and involves utilisation of community resources. The other complications of drain insertion are localised pain (due to the presence of the tube) and poor patient mobility [29–31]. A recent meta-analysis of 12 randomised controlled trials involving 1939 patients randomised to drain (960) versus no drain (979) demonstrated lower morbidity in the no drain group (OR 1.97 (95% CI 1.26-3.10); P=0.003), lower wound infection (OR 2.35 (95% CI 1.22-4.51); P=0.010), and reduced pain 24 hours postsurgery (Standardised Mean Difference 2.30 (95% CI 1.27-3.34); P<0.0001). However they did not show any difference in the intra-abdominal collections and hospital stay [32].

## 8. Conclusion

The vast majority (88.2%) of our LCs were planned as day case, which is higher than those in most specialist units performing LCs. Three-quarters (75.8%) of our planned day case patients were discharged on the same day, which gives a high ‘true day case' rate. The majority of unexpected overnight stays are unavoidable but this may potentially be reduced by a combination of patient selection, stringent preoperative assessment, operation scheduling, and reduction in unnecessary drain insertion. We recommend the reporting of day case surgery should be standardised in the literature to mean same-day discharge; patients who are predicted to have a long or difficult operation should be booked on a morning list to allow adequate recovery time and thus increase the chance of same-day discharge. Patients with BMI >35 or ASA 3 who are routinely scheduled for overnight stay in many units are in our experience often suitable for day case surgery. These measures may increase the overall rate of true day case surgery for LC and have a significant cost benefit by reducing the added bed pressures and financial costs of overnight or further hospital stay.

## Figures and Tables

**Table 1:** Patient demographics: TDC versus UOS.

	**Total (N=438)**	**TDC (n=332)**	**UOS (n=106)**	**P value**
Age (years)	51.0±15.6	49.8±15.6	54.7±15.0	**0.005**

Female gender	347 (79.2%)	269 (81.0%)	78 (73.6%)	0.129

BMI (Kg/m^2^)				
Mean ± SD	29.9±5.5	29.8±5.4	30.5±5.9	0.256
<18.5	2 (0.5%)	1 (0.3%)	1 (0.9%)	0.426
18.5-24.9	67 (15.3%)	56 (16.9%)	11 (10.4%)	0.122
25.0-29.9	164 (37.4%)	122 (36.7%)	42 (39.6%)	0.645
30.0-34.9	113 (25.8%)	83 (25.0%)	30 (28.3%)	0.525
≥35.0	92 (21.0%)	70 (21.1%)	22 (20.8%)	1.000

ASA				
1	184 (42.0%)	154 (46.4%)	30 (28.3%)	**0.001**
2	232 (53.0%)	163 (49.1%)	69 (65.1%)	**0.005**
3	22 (5.0%)	15 (4.5%)	7 (6.6%)	0.443

**Table 2:** Reasons for unexpected overnight admissions (n=106).

**Reason** **∗**	**Number of Patients (%)**
Long operation over 90 min	19 (17.9%)

Drain	26 (24.6%)

Left recovery after 18:30	21 (19.8%)

Post-operative pain	5 (4.7%)

Intra operative complications (small bowel injury)	1 (0.9%)

Retention of urine	7 (6.6%)

Other reasons (anaesthetic issues and social)	21 (19.8%)

Required ERCP post-op (positive OTC)	6 (5.7%)

*∗*Main reason for unplanned overnight stay as per clinician decision listed in the table. Some patients stayed overnight due to multiple reasons.

**Table 3:** Univariate and multivariate analysis of factors contributing to UOS.

**Parameter **	**TDC (N=332)**	**UOS (n=106)**	**Univariate Analysis**	**Multivariate Analysis**		
				**OR**	**95% CI**	**P value**
Age (>50 years)	155 (46.7%)	68 (64.1%)	**0.001**	2.1	1.15-3.78	**0.015**

Age (>60 years)	97 (29.2%)	42 (39.6%)	0.055			

Age (> 70 years)	35 (10.5%)	18 (17.0%)	0.088			

Female gender	269 (81.0%)	78 (73.6%)	0.129			

BMI ≥ 30 Kg/m^2^	153 (46.1%)	52 (49.0%)	0.655			

BMI ≥ 35 Kg/m^2^	70 (21.1%)	22 (20.7%)	1.000			

BMI ≥ 40 Kg/m^2^	19 (5.7%)	9 (8.5%)	0.361			

ASA 3	15 (4.5%)	7 (6.6%)	0.443			

Mean operation time (mins)	64.9±21.6	82.5±35.4	**<0.001**	1.0	1.00-1.04	**0.017**

Length of procedure (>90 minutes)	43 (12.9%)	38 (35.8%)	**<0.001**			

OTC performed	84 (25.3%)	38 (35.8%)	**0.046**			

OTCs in those who had > 90 minutes procedure	15 (4.5%)	16 (15.1%)	**<0.001**			

Drain insertion	0 (0.0%)	26 (24.5%)	**<0.001**			

Time leaving recovery (after 18:30 hrs)	6 (1.8%)	24 (22.6%)	**<0.001**	15.1	5.41-42.34	**<0.001**

Timing of procedure (PM list)	165 (49.7%)	74 (69.8%)	**<0.001**	2.4	1.30-4.61	**0.005**

Intra-operative complications (bowel injury, etc)	0 (0.0%)	1 (0.9%)	0.242			

Immediate post-operative complications (urine retention, bleeding)	0 (0.0%)	7 (6.6%)	**<0.001**			

Operations by trainee (Registrar)	60 (18.1%)	12 (11.3%)	0.131			

**Table 4:** Complications: TDC versus UOS.

**Complications **	**TDC (n=332)**	**UOS (n=106)**	**P value**
Readmissions	9 (2.7%)	2 (1.9%)	1.000
Retained stones	4	1	
Pain	3	1	
Wound infection	1	0	
Haematoma	1	0	

Total complications	6 (1.8%)	8 (7.5%)	**0.007**
	Wound infections (5), haematoma (1)	Small bowel injury (1), urine retention (7)	

Re-operation	0 (0.0%)	0 (0.0%)	1.000

Bile duct injury/bile leak	0 (0.0%)	0 (0.0%)	1.000

Conversion to open	0 (0.0%)	0 (0.0%)	1.000

30-day mortality	0 (0.0%)	0 (0.0%)	1.000

## Data Availability

The data is stored in Hinchingbrooke Hospital computer drive and can be made available on request.

## Appendix

### Day Case Discharge Criteria

*Discharge*

**Table:**

**OUTCOMES AND DISCHARGE CRITERIA**	**MET**	**If NO please give details**
(i) Pain controlled with or without analgesia	□	
(ii) Patient made aware of care required post discharge	□	
(iii) Patient consent scanned and returned	□	

**Table:**

BP, pulse and blood loss stable and within normal range	**YES**	**NO**	**N/A**
Blood loss	□	□	
Tolerable nausea and no vomiting	□	□	
Passing urine normally if relevant	□	□	
Taken food and fluids	□	□	□
Mobile without feeling faint	□	□	□
IV cannula removed	□	□	□
Dressing clean and dry (please state types of dressing supplied)	□	□	□
Pressure areas checked	□	□	□
Patient safe on crutches	□	□	□
Post-op instructions and advise sheet given	□	□	□
Medication given and usage explained	□	□	□
Patient aware of re OPA_________/Details to be posted	□	□	□
Medical/Self certificate	□	□	□
Discharge letter: GP □ Practice nurse □ District nurse □	□	□	□
Day surgery only: Escort home and career to stay overnight	□	□	□
Social support (if yes, describe)	□	□	□
Telephone follow-up arranged for morning	□	□	□
Did Doctor see the patient before discharge?	□	□	□
Procedure specific protocol completed	□	□	□

**Table:**

**DISCHARGE DATE:**	**TIME:**	
**NAME**	**SIGNATURE**	**DESIGNATION**

I agree I am fit for discharge. Signature:……………………………………………

I have been informed of my procedure and after care. Signature:……………………………………………
